# Recent Advances in Magnesium–Magnesium Oxide Nanoparticle Composites for Biomedical Applications

**DOI:** 10.3390/bioengineering11050508

**Published:** 2024-05-17

**Authors:** Abbas Saberi, Madalina Simona Baltatu, Petrica Vizureanu

**Affiliations:** 1 Department of Materials Engineering, South Tehran Branch, Islamic Azad University, Tehran 1777613651, Iran; 2 Department of Technologies and Equipments for Materials Processing, Faculty of Materials Science and Engineering, Gheorghe Asachi Technical University of Iaşi, Blvd. Mangeron, No. 51, 700050 Iasi, Romania; peviz2002@yahoo.com

**Keywords:** magnesium, magnesium oxide nanoparticles, composite, biological characteristics, mechanical properties, corrosion behavior

## Abstract

Magnesium (Mg) is considered an attractive option for orthopedic applications due to its density and elastic modulus close to the natural bone of the body, as well as biodegradability and good tensile strength. However, it faces serious challenges, including a high degradation rate and, as a result, a loss of mechanical properties during long periods of exposure to the biological environment. Also, among its other weaknesses, it can be mentioned that it does not deal with bacterial biofilms. It has been found that making composites by synergizing its various components can be an efficient way to improve its properties. Among metal oxide nanoparticles, magnesium oxide nanoparticles (MgO NPs) have distinct physicochemical and biological properties, including biocompatibility, biodegradability, high bioactivity, significant antibacterial properties, and good mechanical properties, which make it a good choice as a reinforcement in composites. However, the lack of comprehensive understanding of the effectiveness of Mg NPs as Mg matrix reinforcements in mechanical, corrosion, and biological fields is considered a challenge in their application. While introducing the role of MgO NPs in medical fields, this article summarizes the most important results of recent research on the mechanical, corrosion, and biological performance of Mg/MgO composites.

## 1. Introduction

Collagen fibers with a hydroxyapatite (HAP) crystalline matrix form the natural components of bone and are considered as porous biological nanocomposites. Collagen fibers (organic phase) are flexible materials with high toughness, and HAP crystals (inorganic phase) are brittle materials with high hardness. The combination of these two materials with different properties turns bone into a natural biological nanocomposite [1].

Typically, metals such as platinum (Pt), titanium alloys, and stainless steels, which are also compatible with the body, are used as implants in orthopedic surgery [2,3,4,5,6,7,8]. Secondary surgery to remove the implant, which causes physical and mental pain for the patient, as well as the phenomenon of stress shielding on the tissue surrounding the implant, was always a challenge for researchers [9,10]. But the new generation of implants is biodegradable: as tissues heal, the biodegradable material dissolves and forms a harmless oxide, which is excreted in urine. The group of biodegradable metals includes Mg, zinc (Zn), and iron (Fe) [11]. A summarized quantitative comparison of some physical and mechanical properties of common biodegradable metals, in addition to the characteristics of natural bone tissues, is given in Table 1.

**Figure 1:**
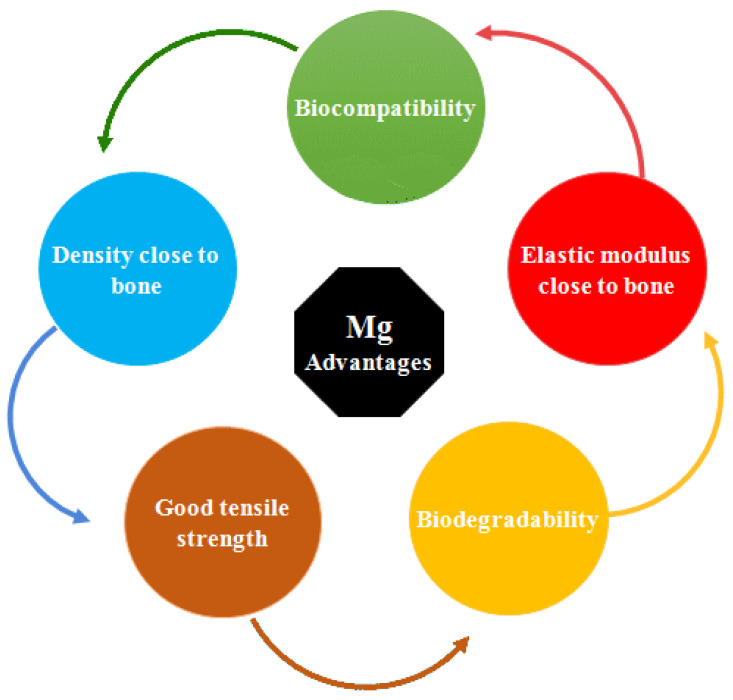
Advantages of Mg metal for orthopedic applications.

Compared to Fe and Zn, Mg has the closest elastic modulus to natural bone at 41–45 GPa. This parameter avoids the phenomenon of stress shielding in the tissues surrounding the Mg implant [19,20,21]. In addition, Mg with a density of 1.7 gr/cm^3^ has the closest density among metals to natural bone with a density of 1.8–2.1 gr/cm^3^ [11]. The benefits of Mg for orthopedic applications are shown in Figure 1. All of these similarities make Mg a suitable candidate for orthopedic applications. Mg is the second most abundant intracellular cation and is known as an important and effective cofactor in more than 300 types of enzymatic reactions, including energy metabolism and protein and nucleic acid production [22,23,24]. Scientific evidence indicates that Mg in the body is distributed in such a way that half is found in soft tissues and half in the bones. The recommended intake of Mg is about 240 to 420 mg/day, which is about 17 to 50 times more than the intake of iron and zinc (15 mg) and about 70 times more than that of strontium and manganese (about 5 mg). Mg deficiency can cause weakness, tremors, seizures, and heart rhythm disorders. Taking extra Mg through medication will lead to hypomagnesaemia, leading to chronic kidney failure and low blood pressure [24,25]. Not long after the discovery of Mg by Sir Humphrey Davy in 1808, the idea of making biodegradable Mg implants was proposed. Edward C. Haas, as a physician, was the first to use Mg wires to stop bleeding in the form of a ligature [26]. However, the main problem of Mg is its low corrosion resistance and rapid degradation rate in human body fluids, which has limited its use as an implant material in medicine. Such rapid and uncontrolled corrosion significantly reduces the mechanical properties of the biological implant and leads to premature failure [27,28]. During body movement, bones are subjected to complex stresses, including shear, tensile, compressive, and tensional stresses. If the implants are in a load-bearing position, they will experience more pressure; all of this necessitates the consideration of mechanical parameters in biological terms. The mechanical and degradation behavior of Mg are seriously affected by the presence of alloying elements and reinforcing materials [21,29,30]. As a result, the determination of alloying elements and reinforcing particles in Mg-based composites is of particular importance. Nanoparticle-reinforced composites have been investigated by many researchers worldwide in recent years due to their promising properties for a large number of functional and structural applications. Reducing the size of the reinforcing phase to the nanoscale means that the interaction of particles with dislocations becomes of considerable importance and, alongside other reinforcing effects commonly found in conventional metal-matrix composites, leads to significant improvements in mechanical properties. In addition, it has been found that it can accelerate the activation of effective biological mechanisms. Recently, Mg NPs have been considered as reinforcements in Mg-based composites due to their unique physicochemical and biological properties, including biocompatibility, biodegradability, non-toxicity of degradation products, and suitable bioactivity with the surrounding tissue. Although there are review articles that introduce the intrinsic properties of MgO NPs and their applications, to date, we have not found any review articles that specifically and comprehensively study the presence of MgO NPs as reinforcements in Mg-based composites for orthopedic applications. Therefore, while introducing MgO NPs and its applications in biomedical science, the latest experimental research on the effectiveness of MgO NPs as reinforcements on the mechanical, corrosion, and biological behavior of Mg-based composites is discussed.

## 2. Magnesium Oxide Nanoparticles (MgO NPs)

**Figure 2:**
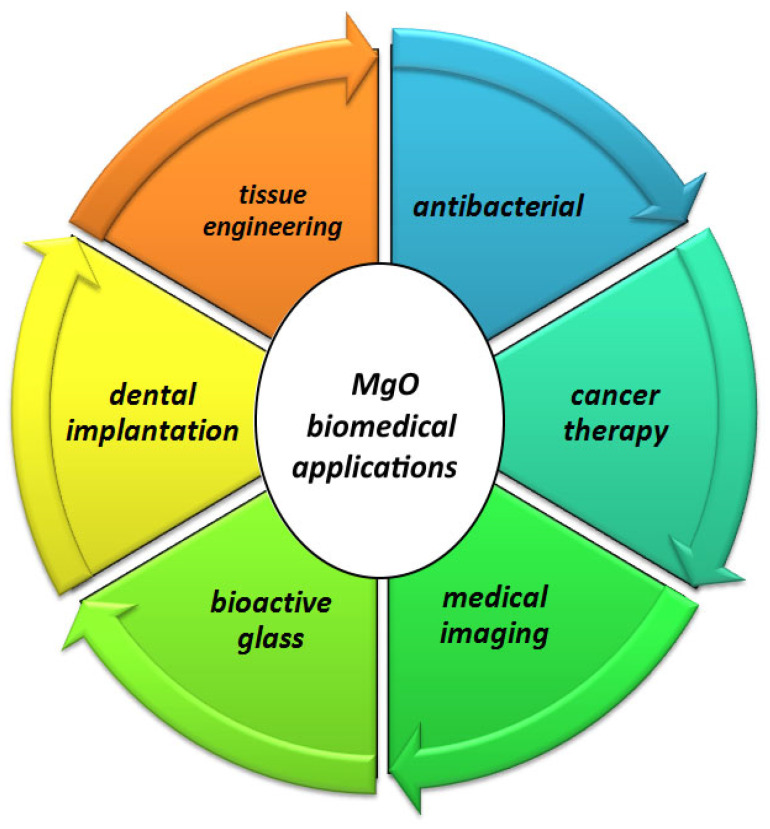
Diverse functionalities of MgO for biomedical applications.

The discovery of nanoscale materials creates new opportunities to expand research into innovative nanosystems and the production of nanocomposites. Many metal oxide nanoparticles have many advantages in the medical field, including MgO NPs. They have antibacterial and anti-cancer properties. MgO NPs are used in the fabrication of biosensors, in cancer diagnosis, and in treatment planning consultation using medical imaging due to their active catalytic properties, high reactivity, and high absorption capacity. Bioactive glass is being developed for applications in surgery, dentistry, bacterial inhibition, bone repair, and tissue engineering. Due to their many properties, such as being antibacterial, anti-cancer, biocompatible, non-toxic, biodegradable, and low-cost, research results support the addition of MgO NPs to a variety of useful compounds [31,32,33]. In the Figure 2, a graphic of the uses of MgO for medical applications is shown. In this section, the most important intrinsic properties of MgO NPs in medical applications are briefly mentioned.

***MgO NPs as an antibacterial:*** Previously, humans used antibiotics to eliminate harmful bacteria. But due to the unnecessary use of antibiotics, bacteria have become resistant to a large number of antibiotics over time [34,35]. Recently, inorganic antimicrobials are increasingly used for decontamination and the prevention of biodegradation. MgO, calcium oxide (CaO), and ZnO exhibited strong antibacterial activity. MgO and CaO powders show significant antibacterial effects on both Gram-positive and Gram-negative bacteria. ZnO powder inhibited the growth of Gram-positive bacteria more strongly than Gram-negative bacteria [36]. MgO NPs are a metal oxide with antibacterial properties. Its properties depend on its shape and size. Nano dimensions for MgO provide better antibacterial activity against *E. coli* and *S. aureus* bacteria. MgO NPs have dose-dependent antibacterial activity. MgO NPs can be metabolized well in the body compared to heavy metal oxide nanoparticles (Ag and Zn), so it is easy to remove degraded ions. MgO NPs show unique antibacterial properties against several common food pathogens. Their contact with bacterial cells leads to cell membrane leakage and induces oxidative stress and cell death [37].

***MgO NPs’ role in cancer therapy:*** Cancer is a genetic disease caused by genes that regulate cellular processes, including growth and division. Nanoparticles smaller than 100 nm can interact with proteins, nucleic acids, and lipids inside and outside cells, which can facilitate cancer diagnosis and treatment. MgO NPs induce lung cancer cell toxicity, possibly due to elevated ROS levels when the mitochondrial membrane potential is altered, triggering the apoptotic process and ultimately leading to cell death. Cytotoxicity testing confirmed that the generated nanostructures were not toxic to healthy red blood cells. MgO nanorods have potential applications as a potent chemotherapeutic agent for the rapid detection and identification of all types of cancer [38,39,40].

***MgO NPs’ role in tissue engineering:*** Tissue engineering is the combined use of cells, material engineering methods, and appropriate biochemical agents to enhance or replace biological tissues. Tissue engineering involves using tissue scaffolds to create new living tissue, all of which provide a three-dimensional environment for cell growth and communication. Hickey et al. [41] investigated the effects of adding MgO NPs to poly (L-lactic) and hydroxyapatite. The results showed that MgO NPs enhance the adhesion and proliferation of osteoblasts on PLLA-HA nanocomposite materials. Furthermore, osteoblasts cultured in the supernatant of the degradable nanocomposite showed enhanced proliferation in the presence of Mg, indicating that the increased alkalinity of the solution containing the MgO nanocomposite did not have a toxic effect on the cells.

***MgO NPs’ role in dental implantation:*** Recently, it has been reported that nanomaterials may have new preventive and therapeutic applications in dental caries. Studies show the effectiveness of Mgo NPs in reducing and controlling plaque biofilm, improving the antibacterial properties of dental materials, and restoring primary dental caries. Passos et al. [42] found that toothpaste containing Mg hydroxide can protect tooth enamel against moderate acid corrosion, but not against severe acid corrosion. Therefore, toothpaste containing Mg hydroxide may be a valuable method of reducing the effects of corrosion. Passos et al. [43] reported that MgO NPs have antibacterial and anti-biofilm effects against various microorganisms, including oral bacteria such as the carcinogenic species S. mutans. Two types of bacteria commonly isolated from the human oral cavity are S. mutans and Streptococcus, and they are recognized as the main cariogenic bacteria. Nanoparticles can affect bacteria in a variety of ways, and bacteria are less likely to be resistant to nanoparticles.

***MgO NPs for bioactive glass:*** Bioceramics are artificial materials that have a good biological interaction with human tissues, and for this reason, they are good candidates in medicine for repairing defects and replacing damaged tissues. Among bioceramics, bioactive glasses (BGs) are a sought-after biomaterial in the field of tissue engineering. BGs have the ability to bond with living tissues of the body by forming a layer at their contact surface with living tissues. Reports show that the production of an HA layer at the interface of BGs and biological tissue is the main reason for this connection between them [44]. In the literature, it is seen that some researchers, in addition to CaO from various metal oxides (MO) such as ZnO and MgO in the structure, have used bioactive glasses based on SiO2-CaO-P_2_O_5_. It was determined that BG-MO nanocomposite material can increase the biological activity and antibacterial activity of bioactive glass. Because of the important functions of the element Mg in human bone metabolism, such as osteoblast differentiation and osteogenic gene expression, oxides based on this element are considered suitable substitutes for application in the structure of bioactive glasses to improve biological activity. It should be noted that MgO is introduced into the preparation of BGs in various ways. In recent studies, MgO was added as a new component to the conventional ternary composition (SiO_2_-CaO-P_2_O_5_) [45,46].

***MgO NPs in medical imaging:*** Molecular imaging, a non-invasive method of imaging body tissues, is a relatively new and exciting field of diagnostic imaging that can be described as the identification and measurement of biological processes at the cellular and molecular level within the body. For example, molecular imaging can be used for detailed examination and diagnosis of cardiovascular diseases such as arrhythmia, blood clot formation in blood vessels, and atherosclerosis [47,48,49]. This method allows for rapid diagnosis of a disease and more accurate prediction of the level of disease. One of the most important parameters of magnetic nanoparticles is their size, as their diameter affects the strength of signal amplification [50]. Nanoparticles smaller than 50 nanometers with a lipophilic coating have a strong ability to cross cell membranes, making magnetic nanoparticles around 50 nanometers in diameter an ideal choice for drug delivery and imaging [51,52]. Magnetic nanoparticles improve image contrast and enable higher-resolution scans, enabling more accurate diagnosis and treatment [53]. The magnetic properties of MgO NPs and their ability to remain in the bloodstream for long periods of time have made them ideal contrast agents for MRI [54]. MgO NPs are non-toxic, have no side effects, are biocompatible, and readily penetrate the human body, making their use vast [55].

## 3. Mg/MgO NP Composites

As mentioned, despite the unique properties of Mg that make it an attractive candidate for medical implants, the high degradation rate of Mg and its low mechanical strength under load are serious obstacles for widespread clinical applications. It has been shown that surface polymer coatings, although they improve corrosion resistance and delay degradation, do not cause any serious mechanical effects on implants [28,56,57]. The development of composites is a reliable solution to improve the corrosion and mechanical behavior of Mg [58,59,60,61,62]. In general, reinforcing phases usually belong to the family of carbon or ceramic materials that are added to improve mechanical properties and corrosion resistance. Carbon materials, including carbon nanotubes and graphene nanosheets, have been used as reinforcements in Mg-based composites due to their unique structure and very high Young’s modulus [21,63,64]. Due to the small diameter and very high mechanical hardness of CNTs, if they are densely placed on the surface of the medical instrument, the obtained lotus effect can provide protection against corrosion as well as bacteria and viruses [65,66]. Abazari et al. [27] showed that the continuous distribution of graphene oxide (GO) reinforcement has a positive effect on the microstructure, mechanical properties, and corrosion resistance of the Mg matrix, while the O-containing groups in GO also promote the deposition of the corrosion product layer [27]. However, the non-degradable nature of graphene has limited its application in the human body as implants [67]. The mechanical performance is significantly increased by adding ceramic particles such as SiC and Al_2_O_3_. But it has a negative effect on corrosion resistance [68,69]. Zhang et al. [70] showed that micro galvanic corrosion in AZ91/SiC composites leads to rapid matrix corrosion. Bakkar et al. [69] reported that the corrosion resistance decreases with the non-uniform distribution of Al_2_O_3_. But on the other hand, bioactive ceramics have the ability to improve the performance of Mg alloys. Campo et al. [71] showed that Mg/HA composites have high microhardness but lower compressive strength than the matrix. However, HA has disadvantages such as a low melting point, poor bonding strength between HA/metals, and limitations in preventing the aggregation of HA particles during the manufacturing process, which eventually leads to loosening of the implant [72,73]. To overcome these problems, bioactive ceramics such as TiO_2_, ZrO_2_, and MgO powders, which have higher chemical stability than apatite structures, have been added to the composite matrix to improve the biological activity and mechanical properties and reduce implant corrosion [72,73,74]. MgO has remarkable antibacterial properties and is one of the main components of bioglass. On the other hand, MgO is very suitable as a reinforcement for making biological composites because it can release Mg^2+^ to be incorporated into the human metabolism [75]. It is known that adding a certain amount of MgO nanoamplifiers can significantly improve the performance of the matrix. Useful research is being carried out to find the optimal amount to achieve greater efficacy in terms of mechanical, biodegradable, biological, and antibacterial effects. These are considered separately below.

### 3.1. Mg/MgO NP Mechanical Properties

In the 1960s, researchers discovered that the presence of a second phase in metals increases the elastic modulus and wear resistance and improves other mechanical properties. The properties of metal-matrix composites (MMCs) depend on many factors such as the manufacturing method, chemical composition, and microstructure, including matrix structure, grain size, precipitation behavior, and lattice defects. Regarding the secondary phase, the volume fraction, physical and mechanical characteristics of reinforcements, size and dimensions, and its distribution method and orientation are considered to be effective factors [76,77]. In this section, theories and strengthening mechanisms that can be effective on Mg/MgO composites are introduced separately, and then the results of experimental research are presented.

***The role of morphology of reinforcements:*** Based on the morphology of the reinforcement, MMCs are usually divided into three types of reinforcement— (1) reinforcements with spherical particles (PRMMCs), (2) reinforcements with short fibers (SFMMCs), and (3) reinforcements with continuous fibers (CFMMCs)—as well as cross-linked (IPC) classification. Among these three categories, PRMMCs are considered the most common type of composite due to their balanced combination of strength, stiffness, wear resistance, and isotropic properties. How to evaluate their mechanical performance is relatively simple due to the presence of almost isotropic reinforcing particles [78,79].

***Load transfer mechanism:*** The direct strengthening method transfers the charge from the soft phase to the harder phases, which, in this case, are ceramic nanoparticles. Load transfer from matrix to reinforcement is usually the most common mechanism in reinforcing MMCs. To transfer the load from the matrix to the reinforcement, the elastic modulus of the reinforcement must be higher than the matrix; on the other hand, the load transfer depends on the bond strength between the reinforcement and the matrix, as well as the volume fraction [13,78].

***Residual stresses:*** Indirect strength is caused by thermal and structural mismatches that prevent the movement of dislocations. The applied loads are also affected by the residual stresses created in the MMCs during the manufacturing process. Residual stress is a self-equilibrium stress that exists without an applied stress and is caused by a natural shape mismatch between two parts, regions, or phases. Since MMCs are manufactured at high temperatures, differences in the CTE of the metal matrix and ceramic reinforcement can create large internal stresses in the individual phases of the composite. When external loads are applied, residual processing stresses within the MMC can add to or subtract from the applied stresses and can have significant effects on the composite’s mechanical response [78,80,81].

***Hall Patch effect:*** Due to the difference in the melting point of ceramic reinforcements and the matrix, they can help to reduce the size of grains as nucleators, resulting in smaller grains in nanocomposites. The relationship between grain size reduction and mechanical strength increase was identified as the Hall Patch effect: σy = σ0 + k√d, where σy is the yield strength, σ0 is a constant, k is a material-dependent constant, and d is the grain size [82,83,84]. This relationship was established based on the observation that the grain boundaries prevent the movement of dislocations, and the amount of dislocations inside the grain also affects the stress generation. Therefore, by changing the grain size, the accumulation of dislocations in the grain and thus the yield strength can be affected [13,85].

***Interaction between the second phase and dislocations:*** On the other hand, the distribution of the second-phase particles dispersed in the metal can interact with the mobile dislocations and increase the strength of the material. Second-phase particles can delay dislocation motion in two distinct ways. If the second phase is small or soft, the dislocations will separate the particles and change their shape. The Orowan mechanism can be used to explain the increase in strain hardening in the presence of incoherent deposits in the metal matrix. In this case, the dislocations bypass the particles and pass through them by bending the dislocation line. Due to the formation of dislocation rings around the grains, the accumulation of dislocations occurs, and for this reason, in a precipitate-hardened crystal, the strain hardening increases suddenly and in the early stages of deformation. Most part strength theories in the second phase are based on spherical particles. Significant studies have shown that the presence of MgO particles in the Mg matrix composite can improve the mechanical parameters [85].

Goh et al. [86] synthesized a Mg/1% MgO composite by melt deposition followed by hot extrusion. An increase in the thermal stability, hardness, tensile strength, and modulus of the nanocomposite was observed compared to the matrix. There was also good surface adhesion between Mg and MgO. In the study by Wang et al. [87], MgO (1–4 wt%)/AZ31 honeycomb matrix composites prepared by low-energy milling followed by extrusion were investigated. The MgO/AZ31 composite consists of a coarse-grained zone and a fine-grained zone. The submicron MgO-rich fine-grained zone forms a honeycomb-like structure, and the MgO-free coarse-grained zone is filled within the unit cells. MgO/AZ31 composites show a good combination of strength and elongation. Honeycomb structures can increase strength [87]. Sadoughi et al. [88] investigated the effect of size and the amount of MgO reinforcement on the properties of Mg. They used reinforcing powders in different sizes and volume fractions: 60 µm, 20 nm, and 1.5, 3, and 5 vol. %, respectively. Specifically, Mg powder and reinforcing materials were mixed in a planetary ball mill for 1 h and then produced at 450 °C for 20 min under a pressure of 600 MPa. The results showed that as the reinforcement content increased, the relative density of the prepared samples decreased. In contrast, the microhardness, wear resistance, and compressive strength increased. The hardness of the nanocomposite containing 5% MgO was 14% higher than its micron counterpart. The wear rate of the nanocomposite containing 5%wt MgO was 45% lower than the same micron composite. The compressive strength of the Mg-5MgO nanosample and Mg-5MgO micro sample exceeded that of the Mg sample by 57% and 54%, respectively. In Table 2, the effect of adding MgO on the mechanical parameters of Mg matrix composites is presented in detail.

### 3.2. Mg/MgO NPs’ Corrosion Behavior

**Figure 3:**
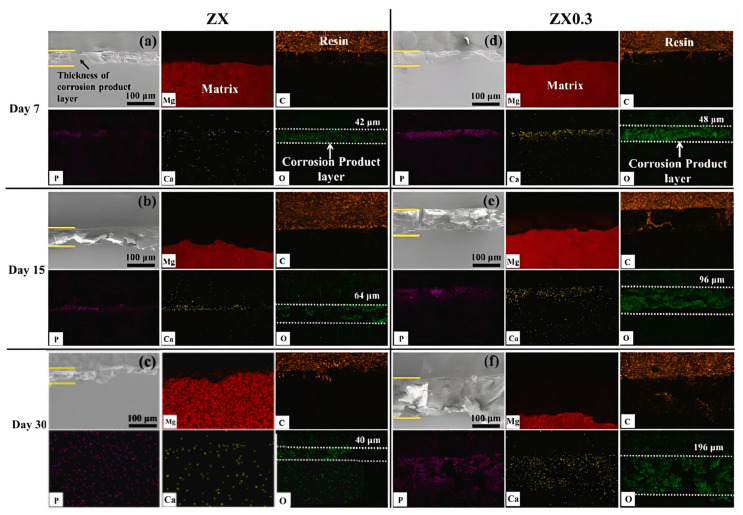
Cross-sectional SEM and EDS micrographs after vitro immersion tests: (**a**–**c**) ZX after 7, 15, and 30 days of immersion; (**d**–**f**) ZX0.3 after 7, 15, and 30 days of immersion [67].

The premature loss of mechanical integrity before the healing of damaged tissues is due to the rapid degradation of Mg. The rapid degradation rate causes the production of excessive amounts of hydrogen and subsequent alkalinization of the environment around the damaged tissue [96]. Therefore, a serious challenge in Mg implants has been to create a rate of degradation proportional to bone growth. The main advantage of using MMCs as a biomaterial is that the mechanical properties and corrosion resistance can be tuned by careful selection of alloy elements and material reinforcements for the metal matrix. Many researchers added various particles of reinforcements to the Mg matrix and evaluated them. Some composites, especially those containing particles of hydroxyapatite (HA) or β-tricalcium phosphate (β-TCP), exhibit severe particle agglomeration due to unfavorable interfaces between Mg/reinforcement and heterogeneous distribution. This leads to severe localized pitting corrosion and a subsequent loss of mechanical properties. To overcome these problems, some bioactive ceramics with high chemical stability (ZrO_2_, TiO_2_, and MgO) are more suitable as reinforcements for the Mg matrix. MgO is one of the main components of bioglass with excellent thermal and mechanical properties. Furthermore, MgO can be completely degraded to produce the same products as Mg in vivo [97]. Zamani Khalajabadi et al. [89] studied Mg/HA/MgO nanocomposites with pure Mg and various amounts of hydroxyapatite and periclase nanoparticles added using the powder metallurgy method. The corrosion resistance of nanocomposites increases from 0.25 kω2 cm^2^ to 1.23 kω cm^2^ by adding MgO at 10%wt. In addition, a decrease in the value has been shown from 27.5 to 12.5 wt %; composite surface corrosion products are primarily Mg(OH)_2_, HA, and CA3 (PO4)_2_. During immersion in the SBF solution, the growth of Mg(OH)_2_ on the surface of the nanocomposites creates a barrier oxide layer between the SBF solution and the substrate and disrupts the corrosion process for some time [89]. Tang et al. [67] fabricated a Mg-3Zn-0.2Ca-0.3MgO (wt.%, ZX0.3) composite by the hot extrusion method for biological applications. The results of the degradation tests showed that the MgO NPs in the composites not only promoted the formation of the Mg(OH)_2_ layer but also effectively prevented crack propagation in the corrosion product layer. It prevents corrosive liquids from penetrating the matrix, significantly increasing the density of the corrosion product layer and increasing its durability. The corrosion rate of the ZX0.3 composite was reduced by 30% to 0.79 mm/year compared to that of the Mg-3Zn-0.2Ca (ZX) alloy. Furthermore, the lower corrosion rate provides a safe environment for cell adhesion and differentiation, thereby improving the biocompatibility of ZX0.3 compared to the ZX alloy [67]. Figure 3 shows SEM and EDS images of cross-sections of the ZX alloy and ZX0.3 composites exposed to SBF solution on different days. By identifying the distribution of O, Ca, and P elements, the thickness of the corrosion product layer can be measured at different times. The distribution of the C element represents the position of the resin used to mount the specimen. A relatively dense Ca-P corrosion product layer formed on the ZX0.3 surface after 7 days of immersion with a depth of 48 μm; the thickness of the corrosion product layer after 15 and 30 days was measured to be 96 and 196 μm. On the other hand, there is a corrosion product layer on the surface of ZX, but the EDS results show that the Ca-P layer is very thin and has low density. Therefore, it cannot exert a good protective effect on the matrix [67]. In Table 3, the effect of MgO addition on the corrosion parameters of Mg matrix composites is presented in detail.

### 3.3. Mg/MgO NPs’ Biological Properties

In bone, where Mg is present in the highest concentrations, Mg cations are located at the edges of apatite minerals and directly influence the size and density of the mineral—an important element contributing to the unique mechanical properties of bone. In addition, these Mg ions indirectly influence mineral metabolism through the activation of alkaline phosphatase. Beyond its cooperative role with HA in maintaining bone health, Mg ions play an important role in mediating the functions of all cells in the body, particularly through the activation of integrins. Divalent Mg^+2^ (and Ca^+2^) ions initiate the conformational activation of integrins for ligand binding by binding to sites on the alpha chain of integrins, thereby leading to cellular functions such as binding, proliferation, and migration [98]. Therefore, incorporating MgO into tissue engineering constructs may improve cell–scaffold interactions. On the other hand, nanotechnology produces different types of nanoparticles that can cause fundamental changes in materials such as small particle size, variable shape, and higher surface-to-volume ratio, as well as biological, mechanical, and physical changes, among others; it seems to be developing more and more in the fields of biomedicine and biomaterials [99]. Among the types of metal oxide nanoparticles, MgONPs have attracted a lot of attention due to their unique biocompatibility, non-toxicity, high stability in harsh conditions, and diverse and significant applications, especially in the field of biomedicine. MgONPs have several useful physicochemical properties, such as high ionic character, large surface area, unusual crystal morphology, and oxygen vacancies, which enable them to easily interact with several biological systems [100,101].

The antibacterial properties of MgO NPs are of interest for medical use. The high pH of MgO NPs (alkaline pH) may contribute to their antibacterial activity [102]. Despite the complexity and unknown antibacterial mechanisms in metal oxides, the main antibacterial mechanisms for metal oxides, including Mg NPs, can be classified as follows: (1) physical damage to the bacterial cell wall as a result of the electrostatic interaction of the sharp edges of nanomaterials with the cell wall membrane; (2) the production of ROS; (3) the entrapment of bacteria in aggregated nanomaterials; (4) the disruption of bacterial glycolysis; (5) oxidative stress; (6) DNA damage; (7) the perturbation of proteins and cell structure, leading to the release and interaction of metal cations and alkaline effects; and (8) metal ion release [103,104,105,106].

**Figure 4:**
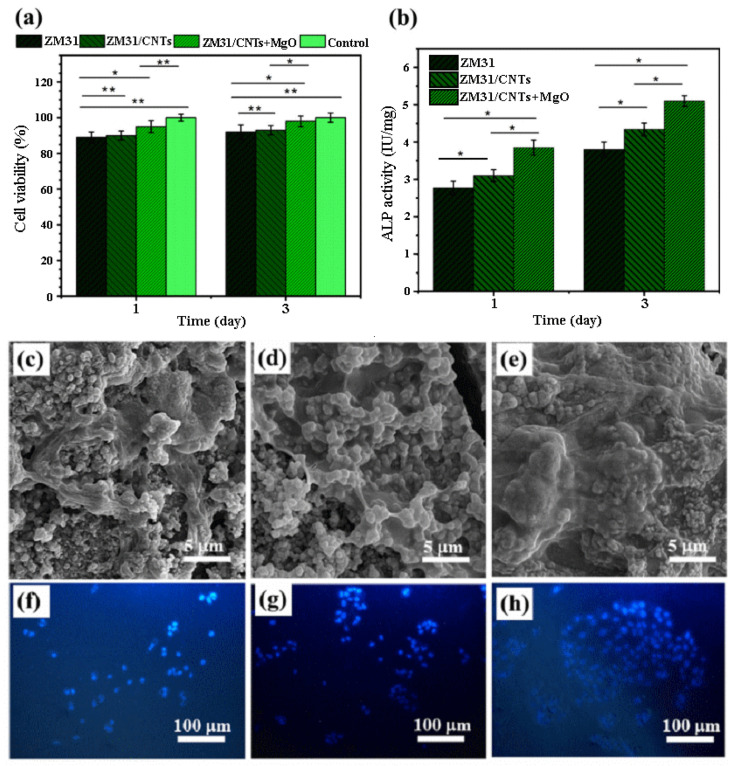
(**a**) Cell viability and (**b**) ALP activity of MG-63 cells cultured for various amounts of time on ZM31 alloys, ZM31/CNTs, and MgO-ZM31/CNT nanocomposites, and SEM images of the morphology and adhesion of these MG-63 cells and fluorescent DAPI staining of these cells grown on (**c**,**f**) ZM31 alloy, (**d**,**g**) ZM31/CNTs and (**e**,**h**) ZM31/MgO-CNT nanocomposites for 3 days (* *p* < 0.05 and ** *p* < 0.01) [90].

In the study by Abazari et al. [90], Mg-3Zn-1Mn(ZM31)/CNTs and ZM31/MgO NP-CNT composites were prepared by semi-powder metallurgy followed by hot extrusion. Cell viability was higher in ZM31/CNTs and ZM31/MgO-CNT composite extracts than in the Mg alloy extract, and the cell activity increased with culture time. These results showed that the surface of MgO-conjugated CNTs significantly improved the biocompatibility of Mg-based composites. Cells cultured on ZM31/MgO-CNT compounds showed stronger ALP staining than cells cultured on Mg alloys, but ZM31/CNTs complexes had lower ALP activity than ZM31/MgO-CNT complexes. These results showed that the response of Mg alloy cells was improved by using CNTs incorporated in MgO. In the same culture time, MG-63 cells cultured on ZM31/MgO-CNT composites showed slightly higher cell attachment than the Mg alloy. This was probably due to faster degradation and alkalinization of the surface, which could have prevented MG-63 adhesion and caused cell membrane disruption due to oxidative damage (Figure 4) [90].

## 4. Summary and Future Road Maps

Mg appears to be an attractive option for fracture fixation implants and temporary stents in orthopedics due to its density and elastic modulus close to bone, as well as its fully biodegradable products. However, serious challenges facing Mg have limited the development of its applications. Among others, we can mention its high rate of degradation and, as a result, the loss of its mechanical properties. It seems that the smart development of Mg-based nanocomposites, in addition to improving the mechanical and corrosion behavior, can have a significant effect on the bioactive performance of the Mg matrix. Metal oxide nanoparticles have a distinct and irreplaceable position in the category of nanomaterials, which today have found a variety of applications due to their unique chemical and physical properties and wide applicability in various fields, including biomedical technology. MgO NPs are distinguished from other metal oxide nanoparticles in terms of biocompatibility, biodegradability, bioactivity, and antibacterial properties. Some research shows that the presence of Mg NPs as a reinforcement in the Mg matrix significantly improves the mechanical performance. To justify this effect, causes and mechanisms such as (1) the role of the morphology of reinforcements, (2) the load transfer mechanism, (3) residual stresses, (4) the Hall patch effect, and (5) interactions between the second phase and dislocations are mentioned in detail in the literature. On the other hand, it has been found that MgO NPs in SBF solution facilitate the formation of Mg(OH)_2_ layers and also increase the thickness of Ca-P surface layers. However, it seems that to prevent pitting and galvanic corrosion, the uniform distribution of the second phase and determining the optimal amount are effective factors. Incorporation of MgO into tissue engineering constructs may improve cell–implant interactions. However, it appears that the cytotoxic effects of MgO NPs on normal cells and living organs should be investigated. In vivo and clinical scientific data, especially in biological and antibacterial aspects, are very limited. Therefore, there is a serious need for comprehensive in vitro and in vivo applied research in order to investigate the effectiveness of MgO NPs as reinforcements of Mg-based composites in order to provide useful results to the scientific community.

## Figures and Tables

**Table 1:** Comparison of the mechanical properties and degradation rates of bone tissues with biodegradable metallic materials.

Tissue/ Material	D (g/cm^3^)	E (GPa)	UTS (MPa)	YS (MPa)	FT (MPa m^1/2^)	ε (%)	MH (HV) (kgf/mm^2^)	UCS (MPa)	D.R	Ref.
Natural bone	1.7–2.1	3–30	35–283	70–100	3–6	1–4	35–40.4	164–200	NBR	[12,13]
Pure Mg	1.74	41–45	135–285	130–250	15–40	5–40	42	65–100	0.8–2.7	[14,15,16]
Pure Fe	7.8	213	300–540	120–150	25–60	37.5	150	560	0.1	[13,14,17]
Pure Zn	7.1	78–121	97–150	21–30	35–120	0.3–2	32–44	30–100	0.1–0.3	[11,18]

D: density; E: Young’s modulus; UTS: ultimate tensile strength; YS: yield strength; FT: fracture toughness; ε: elongation; MH: microhardness; UCS: ultimate compressive strength; D.R: degradation rate; NBR: natural bone remodeling.

**Table 2:** Effect of MgO on mechanical properties of Mg matrix composites.

Materials	Method	Microhardness	0.2%YS (MPa)	UTS (MPa)	UCS (MPa)	FS (%)	Ref.
Mg (99.9% purity)	DMD-HEXT	45 ± 1	126 ± 7	192 ± 5	-	-	[86]
Mg–0.5MgO	DMD-HEXT	47 ± 1	151 ± 3	233 ± 5	-	-	[86]
Mg–0.75MgO	DMD-HEXT	53 ± 1	158 ± 5	213 ± 4	-	-	[86]
Mg–1.0MgO	DMD-HEXT	54 ± 2	169 ± 8	223 ± 8	-	-	[86]
Mg–27.5%HA	PM	-	-	-	237 ± 6	4.2 ± 0.4	[89]
Mg–20%HA–5%MgO	PM	-	-	-	202 ± 11	4.4 ± 0.6	[89]
Mg–12.5%HA–10%MgO	PM	-	-	-	198 ± 9	11.5 ± 2.1	[89]
Mg–5%HA–15%MgO	PM	-	-	-	183 ±14	11.8 ± 1.7	[89]
Mg-3Zn-1Mn	SPM-EXT	51.6 HV	-	-	295.6 ± 20.4	-	[90]
Mg-3Zn-1Mn/CNTs	SPM-EXT	74.5	-	-	404.8 ± 16.1	-	[90]
Mge3Zn-1Mn/MgO-CNTs	SPM-EXT	83.4 HV	-	-	429 ± 15	-	[90]
AZ31	LEM-EXT	-	231.9	332.8	-	17.6	[87]
1MgO/AZ31	LEM-EXT	-	246.2	344.1	-	21.5	[87]
2MgO/AZ31	LEM-EXT	-	253.1	351.9	-	18.5	[87]
4MgO/AZ31	LEM-EXT	-	265.3	361.2	-	15.4	[87]
Pure Mg	ECAE	41.7 ± 2.3	-	-	135	2.56	[91]
Mg + 10% MgO	ECAE	44.9 ± 0.5	-	-	185	1.80	[91]
Mg + 20% MgO	ECAE	49.3 ± 2.2	-	-	172	1.93	[91]
Mg + 30% MgO	ECAE	52.6 ± 2.5	-	-	160	2.23	[91]
Mg-3Zn-0.2Ca	As-extruded	53.89 ± 1.98		-	378.46 ± 7.35	16.84 ± 0.81	[92]
Mg-3Zn-0.2Ca/0.6MgO	As-extruded	58.29 ± 2.15		-	409.12 ± 4.86	14.10 ± 0.41	[92]
Mg-3Zn-0.2Ca/0.6MgO	1-pass ECPed	60.30 ± 3.08		-	362.35 ± 9.33	26.31 ± 0.37	[92]
Mg-3Zn-0.2Ca/0.6MgO	4-pass ECPed	67.58 ± 2.31		-	379.06 ± 14.20	34.05 ± 0.85	[92]
Mg-3Zn-0.2Ca/0.6MgO	8-pass ECPed	71.55 ± 2.80		-	405.77 ± 12.51	34.18 ± 0.99	[92]
Mg-0.3Sr-0.3Ca	VSC, HEXT	50 ± 2.5	174 (TYS) 68 (CYS)	233	300	7.4 (TFS) 15.2 (CFS)	[93]
Mg-0.3Sr-0.3Ca/0.2GNPs	VSC, HEXT	53 ± 3	213 (TYS) 90 (CYS)	235	303	10.2 (TFS) 16.9 (CFS)	[93]
Mg-0.3Sr-0.3Ca/0.2GNPs + 1.5MgO	VSC, HEXT	63 ± 3	224 (TYS) 96 (CYS)	239	330	13.8 (TFS) 18.3 (CFS)	[93]
Mg pure	PM-HEXT	-	127 ± 5	205 ± 4	-	-	[94]
Mg-0.1MgO	PM-HEXT	-	141± 8	213 ± 4	-	-	[94]
Mg-0.2MgO	PM-HEXT	-	146 ± 8	206 ± 8	-	-	[94]
Mg-0.3MgO	PM-HEXT	-	148 ± 6	208 ± 8	-	-	[94]
Mg-0.4MgO	PM-HEXT	-	137 ± 3	192 ± 4	-	-	[94]
Pure Mg	PBM	46.54	-	-	109.7	-	[88]
Mg-1.5vol.% MgO- micro	PBM	49.28	-	-	119.4	-	[88]
Mg-3vol.% MgO- micro	PBM	50.18	-	-	127.6	-	[88]
Mg-5vol.% MgO- micro	PBM	56.3	-	-	141.5	-	[88]
Mg-1.5vol.% MgO- nano	PBM	55.96	-	-	136.9	-	[88]
Mg-3vol.% MgO- nano	PBM	59.88	-	-	152.3	-	[88]
Mg-5vol.% MgO- nano	PBM	65.26	-	-	168.4	-	[88]
Mg-3Zn-0.2Ca	HSMC-HA-HEXT	-	257.4	298	-	16.5	[95]
Mg-3Zn-0.2Ca	HSMC-HEXT	-	243.5	289	-	20	[95]
Mg-3Zn-0.2Ca-0.1MgO	HSMC-HEXT	-	263.7	301	-	19.2	[95]
Mg-3Zn-0.2Ca-0.2MgO	HSMC-HEXT	-	277.6	309	-	15.1	[95]
Mg-3Zn-0.2Ca-0.3MgO	HSMC-HEXT	-	289	317	-	14.6	[95]
Mg-3Zn-0.2Ca-0.5MgO	HSMC-HEXT	-	300	329	-	14.1	[95]

YS: yield strength; UTS: ultimate tensile strength; UCS: ultimate compressive strength; FS: fracture strength; DMD: disintegrated melt deposition technique; HEXT: hot extrusion; LEM: low-energy milling; EXT: extrusion; PM: powder metallurgy; ECAE: equal channel angular extrusion (ECAE); VSC: via stir casting; TFS: tensile fracture strength; CFS: compressive fracture strength; PBM: planetary ball mill.

**Table 3:** Effect of MgO on corrosion parameters of Mg-based composites.

Samples	Method	Electrolyte	CR (mm/year)	Ecorr (V/SCE)	Icorr (µA/cm^2^)	Ref.
Mg–27.5%HA	PM	SBF	4.28	−1.4873	187.4	[89]
Mg–20%HA–5%MgO	PM	SBF	4.65	−1.4346	203.6	[89]
Mg–12.5%HA–10%MgO	PM	SBF	1.06	−1.2582	46.8	[89]
Mg–5%HA–15%MgO	PM	SBF	1.94	−1293.8	85.2	[89]
Mg-3Zn-1Mn	SPM-EXT	SBF	2.77	−1.53	121.34	[90]
Mge3Zn-1Mn/CNTs	SPM-EXT	SBF	2.32	−1.47	101.56	[90]
Mge3Zn-1Mn/MgO-CNTs	SPM-EXT	SBF	1.98	−1.43	86.73	[90]
Pure Mg	ECAE	NaCl solution	1.66	−1.4173	201	[91]
Mg + 10% MgO	ECAE	NaCl solution	14.18	−1.3881	23.1	[91]
Mg + 20% MgO	ECAE	NaCl solution	28.21	−1.4442	64.6	[91]
Mg + 30% MgO	ECAE	NaCl solution	39.21	−1.5442	84.6	[91]
Mg-0.3Sr-0.3Ca	VSC, HEXT	SBF		1.832	7.373	[93]
Mg-0.3Sr-0.3Ca/0.2GNPs	VSC, HEXT	SBF		1.776	6.980	[93]
Mg-0.3Sr-0.3Ca/0.2GNPs + 1.5MgO	VSC, HEXT	SBF		1.800	9.279	[93]
Pure Mg	PBM	NaCl solution	2.168	−2196.71	58.6	[88]
Mg-1.5vol.% Mgo—micro	PBM	NaCl solution	8.897	−1921.06	131.6	[88]
Mg-3vol.% Mgo—micro	PBM	NaCl solution	19.679	−1336.71	321.8	[88]
Mg-5vol.% Mgo—micro	PBM	NaCl solution	28.456	−1314.16	461.7	[88]
Mg-1.5vol.% Mgo—nano	PBM	NaCl solution	5.642	−2103.67	89.5	[88]
Mg-3vol.% Mgo—nano	PBM	NaCl solution	12.714	−1719.83	182.1	[88]
Mg-5vol.% Mgo—nano	PBM	NaCl solution	17.561	−1695.12	241.3	[88]
Mg-3Zn-0.2Ca	HSMC-HA-HEXT	SBF	6.44 ± 0.65	−1.749	84.9	[95]
Mg-3Zn-0.2Ca	HSMC-HEXT	SBF	4.45 ± 0.21	−1.729	40.4	[95]
Mg-3Zn-0.2Ca-0.1MgO	HSMC-HEXT	SBF	3.92 ± 0.45	−1.669	33.5	[95]
Mg-3Zn-0.2Ca-0.2MgO	HSMC-HEXT	SBF	3.10 ± 0.2	−1.659	16.4	[95]
Mg-3Zn-0.2Ca-0.3MgO	HSMC-HEXT	SBF	3.55 ± 0.42	−1.689	24.5	[95]
Mg-3Zn-0.2Ca-0.5MgO	HSMC-HEXT	SBF	5.40 ± 0.3	−1.729	69.3	[95]

PM: powder metallurgy; SPM: semi-powder metallurgy; ECAE: equal channel angular extrusion; VSC: via stir casting; PBM: planetary ball mill; HSMC: high-shear melt conditioning; HA: homogenizing annealing; HEXT: hot extrusion.

## Data Availability

Not applicable.
